# Voices of NGOs Supporting the First Master’s Degree Program in Ethology and Human-Animal Interactions in Romania: An Exploratory Qualitative Analysis

**DOI:** 10.3390/ani11041091

**Published:** 2021-04-11

**Authors:** Alina Simona Rusu, Adriana Dalila Criste, Daniel Severus Dezmirean

**Affiliations:** 1 Faculty of Psychology and Sciences of Education, Babeș-Bolyai University, 400029 Cluj-Napoca, Romania; 2 Faculty of Animal Science and Biotechnology, University of Agricultural Sciences and Veterinary Medicine, 400372 Cluj-Napoca, Romania; adriana.criste@usamvcluj.ro (A.D.C.); ddezmirean@usamvcluj.ro (D.S.D.)

**Keywords:** higher education professional program, human-animal interactions, animal protection NGOs, wildlife conservation NGOs, qualitative needs analysis, community engaged university

## Abstract

**Simple Summary:**

The objective of this exploratory study was to investigate through a qualitative thematic analysis the reflections of six animal protection and wildlife conservation non-governmental organizations (NGOs) in Romania regarding the development of the first specialized master’s degree program in ethology and human-animal interaction in the country. The two research questions addressed in the study were (1) What are the needs of the participating NGOs that could be addressed by the first professional master’s program in ethology and human-animal interactions in Romania? (2) What are the expectations regarding the roles of the graduates of the program on the collaboration with the NGOs in addressing common problems in the area of human-animal interactions in Romania? The qualitative content analysis of the written reflections allowed the identification of two themes, which provided us valuable insights regarding the curriculum offerings and the pedagogical strategies that could respond to the expressed expectations. The first theme refers to the concerns expressed by the representatives of the NGOs over the animal welfare and human-animal interactions in Romania, which included seven categories of codes: concerns over stray animals, lack of education of general population on animal welfare and interactions with animals, maltreatment of animals, human-animal conflicts, lack of professional specializations in ethology and human-animal interactions, concerns over common global issues, and concerns over national nature conservation. The second identified theme refers to the expectations regarding the roles of the graduates of the master’s degree program, and it includes the following four categories: agents for change towards a responsible community, problem solvers, public policy-makers, and providers of competence-based expertise.

**Abstract:**

This study aims to explore through a qualitative thematic analysis the reflections of six animal protection and wildlife conservation NGOs in Romania regarding the development of the first specialized master’s degree program in ethology and human-animal interaction (EHAI), in relation to the identified needs based on their experience in the field, as well as their expectations in terms of collaboration with the graduates of such a program in addressing the common problems in the areas of animal welfare and human-animal interactions (HAIs). The qualitative content analysis of the written reflections allowed the identification of two themes, which provided valuable insights regarding the curriculum offerings and the pedagogical strategies that could respond to the expressed needs and expectations. The first theme included seven categories of concerns expressed by the NGOs over the animal welfare and HAIs in Romania, i.e., concerns over stray animals, lack of education of general population on HAI and animal welfare, maltreatment of animals, human-animal conflicts, lack of professional specializations in HAI, concerns over common global issues, and concerns over national nature conservation. The second theme refers to the expectations regarding the roles of the graduates of the EHAI master’s program, and it includes four categories of codes: agents for change towards a responsible community, problem solvers, public policy-makers, and providers of competence-based expertise. The inclusion of Service-Learning as signature pedagogy in the EHAI program, in connection with the One Welfare approach, is discussed in relation to the needs expressed by the representatives of the NGOs.

## 1. Introduction

The development of academic interest in human-animal interactions (HAIs) is reflected in the increasing number of research publications in the field of anthrozoology, as well as by the number of Higher Education programs around the world. The diversity of the interactions shared between humans and animals (species with whom humans interact more regularly, such as companion animals, species involved in animal-assisted interventions, farm animals, animals used for sports, captive wildlife from zoos and aquariums, wild animals, etc.) is reflected in the diversity of disciplines addressing aspects of these interactions not only within the field of anthrozoology but also within other fields, such as human and veterinary medicine, psychology, sciences of education, biology, geography, anthropology, political sciences, economic sciences, etc. [1,2,3,4].

In terms of competencies-based curricula, the Higher Education degree programs in the field of animal welfare, ethology, and anthrozoology that currently exist in the European space (and around the world) are usually highly connected with the professions of ethologists, conservation specialists, animal therapists, and animal behaviorists, which are framed within national and EU legislation (in the case of the EU countries), as well as regulations that are implemented by national boards of professionals. A recent synthesis of the current situation of university centers and programs for animal studies and human-animal interactions indicates that the interest of animal issues among university academics has risen in part because of the tremendous growth of the animal protection movement and the legislative frames and declarations at national, regional, and international levels [5]. 

The collaboration between Higher Education Institutions (HEIs) and the representatives of the community, such as non-governmental associations (NGOs) and other institutions (e.g., shelters, farms, zoos, and aquariums), can offer important insights on the community needs and on the applicability of research and education to real-world scenarios [2,4,6,7]. One of the most common and simple definitions of an NGO is “…an organization, that is not owned, directed by of affiliated to any governmental organization, which espouses a variety of ideologies and causes and operates on a non-for-profit basis” [8] cited in ref. [9]. Klugman [8], in her publication “The Role of NGOs as agents for change,” discusses the NGOs’ participation as an essential element to good policy-making and implementation, including here aspects of educational systems’ values, missions, and policies. The World Association of Non-Governmental Organizations (WANGO) recommends that any form of cooperation between NGOs and other organizations and institutions, including universities, should be based on the following four principles: (1) missions are consistent with each other; (2) collaboration is made on the basis of shared values and for the good of society; (3) there are equitable and mutual benefits; and (4) the partnership is committed to financial transparency and the sharing of information, ideas, and experiences [9,10]. The areas of cooperation between NGOs and HEIs refer usually to the following elements [9]: research centers (with emphasis on current rising issues and solving societal needs through applied and participatory research); Service-Learning (an experiential form of pedagogy, often included within the category of Humane Education, which connects learning with service in the community by offering the students opportunities for meaningful reflection [11,12,13,14]); courses, seminars, conferences, and other types of joint educational programs and awareness events; field education placements for students, etc. 

Organizations that have a central task in connecting humans to animals and the environment are expected to be familiar not only with the existent legislative frames in animal protection and wildlife conservation but also with the curricular offerings of the academic programs (including the emerging ones) in charge with the formation of specialists addressing the diversity of animal issues [4]. Anthrozoology academic programs are currently functioning at several universities and colleges around the world (the United States, Canada, the United Kingdom, New Zeeland, Sweden, Spain, Germany, Austria etc.; [5]). Among them, some programs are research oriented, with inter- and multi-disciplinary approaches (e.g., the Interdisciplinary Master Program in Human-Animal Interactions at Veterinary Medicine University, Vienna, Austria), and others are specialized professional master’s degree programs (e.g., Master’s degree in Ethology and Companion Animals, Barcelona Autonomous University, Spain). Even though the conditions of working together of HEIs with NGOs may vary substantially in different countries [9], one can easily infer that the cooperation between HEIs and animal protection and environmental NGOs, such as the professional-based and community engagement offerings for students (i.e., field education, Service-Learning, volunteering, employability resources) and for the academics (i.e., research opportunities, transfer of knowledge applied to real-world needs), is facilitated by the existence of specialized degree programs in animal behavior, animal welfare, and human-animal interactions.

Romania is one of the EU countries with no specialized degree programs in anthrozoology, ethology, and animal welfare, although subjects such as ethology, ethopathology, and animal welfare (mainly farm and wild animals) can be found in the curricular offerings of veterinary medicine, biology, and ecology, within the graduate and undergraduate programs. The animal-assisted interventions subject is currently offered at only one university in Romania (having a duration of one semester), within a master’s degree in Special Education, Faculty of Psychology and Sciences of Education, Babes-Bolyai University.

For the first time in the academic history of the country, a master’s degree program in Ethology and Human-Animal Interactions (EHAI) is currently being developed in English language at the School of Animal Science and Biotechnology, University of Agricultural Sciences and Veterinary Medicine in Cluj-Napoca. The master’s degree program will have duration of 2-years, and it is expected to launch in July 2021. The university is one of the state HEIs in Romania, and it has been recently ranked on the 18th position among the 102 Romanian HEIs included in the Web Ranking of the World Universities in 2021 [15]. Founded in 1906, initially as an Agricultural Sciences University, in 1995, the university had included veterinary medicine as a distinct school with several departments [16].

Inspired by the structure of similar successful degree’s programs existing in Europe and around the world, the team of the EHAI program is an interdisciplinary one, being composed of academic experts in from the School of Animal Science and Biotechnology, School of Veterinary Medicine (University of Agricultural Sciences and Veterinary Medicine in Cluj-Napoca), and the School of Psychology and Sciences of Education (Babes-Bolyai University, Cluj-Napoca). The collaboration between the two HEIs in the field of human-animal studies was consolidated over the last 10 years through several research projects and educational ones, such as a distance learning postgraduate program in animal-assisted interventions (AAIs) for persons with special needs (the only existing academic training in the field of AAI in Romania) and by organizing community-oriented campaigns involving students for the promotion of the responsible ownership and optimal human-animal interactions.

The planning of this specialized master’s degree in Romania in this particular moment is motivated by a series of favorable factors, as they follow: the constant signals coming from the animal protection and wildlife conservation NGOs in Romania regarding the crucial demand for academically trained experts in animal welfare and behavior, and the recent legislative changes in the area of animal welfare, including a law proposal for the profession of ethologist/animal behaviorist. Particularly, we consider that this law proposal opens tremendous opportunities of the HEIs in Romania to offer professional graduate and postgraduate degrees supporting the emergence of this profession at national level. Some examples of problematic human-animal interaction cases in Romania are presented below, which were highly reflected in the national and international media. In all these cases, several national NGOs in the field of animal protection and animal welfare manifested immediate reactions by direct implication in solving the problematic situations and by expressing the need for more efficient structures in charge with the reinforcement of the existing legislative frame in animal protection and cruelty prevention in Romania.

Among other countries, Romania is often cited as a problematic country in global surveys and reports on the growing population of stray animals in urban areas, as well as negative human-animal interaction incidents [17,18], such as attacks of people by dogs in public parks and forms of intentional and non-intentional abuse towards animals (e.g., dogs, cats, and horses). Despite some cases dramatically reported by mass media, e.g., a case of a little boy killed by a group of dogs in Bucharest near a public park in 2013, and despite the efforts of many NGOs and of some local municipal structures to promote adoption of stray animals, to implement trap-neuter-return programs and to prevent the abandonment of the animals, the public and private shelters in Romania are still overcrowded [18]. The problematic issues refer not only to the interactions with dogs and cats but also to inadequate management of livestock transportations. In line with this, a recent case is represented by the disaster recorded in November 2019 in Constanta, Romania, when a cargo ship transporting 14,000 live sheep capsized shortly after leaving the port of Midia. Among the team members involved in the 5-days rescue mission of the agonizing sheep were veterinarians of one NGO in Romania, with expertise in the field of rehabilitation of wildlife and animal protection. The event has raised a lot of public attention alongside with critical questions about the animal welfare conditions on the vessels transporting live animals by sea (note: as indicated by the media reports, Romania is the European Union’s largest exporter of live sheep to the Middle East; [19]). It is important to mention that, following the rescue mission of the few surviving sheep, the reflections posted on the social media by one of the involved veterinarians on the emotional impact of the event, indicate the need for addressing the self-care and compassion fatigue of the persons involved in animal-oriented professions in Romania, in line with the existing curriculum offerings and therapeutic programs around the world [20,21,22,23]. 

As already stated above, the particular timing of developing the EHAI master’s degree program is also marked by recent promising changes made by the Romanian Government in the area of animal welfare, particularly in the last 2 years (2019–2020). The first version of the national law on animal protection in Romania (National Law 205) dates from 2004, i.e., the same year in which Romania received the political confirmation of the EU accession negotiations, the accession taking place in 2007, January 1. Several modifications to the original version of the law in accordance to the EU legislations and existing treaties have been made ever since [24,25]. In accordance with the EU recommendations for Romania and of those issued by the Animal Protection Index [18], the last 2 years of the Romanian Government interest toward positive changes in the area of animal welfare is characterized by important legislative projects that are currently implemented and/or in the process of being analyzed at level of specific national committees. One of these refers to the foundation of the Animal Police structure (Government Ordinance no. 175/2020, [26]), which is based in the following official considerations: the obligation of the State to set up a complex of animal protection measures so that their health and well-being are ensured and they can quickly benefit from specialized public shelter services in the event that they are subjected to dangerous situations, to ensure services involving emergency veterinary care, including surgery, transport, accommodation, feeding, deworming, vaccination, and control of animal diseases and to establish efficient measures to enable strategic decisions to be taken at the level of the Romanian Government regarding the management of animal abuse or cruelty, following the model provided by existing similar structures in other countries that are members of the European Union. The second promising change made by the Romanian Government is the recent National Law Project (no. 210/2020, [27]) for the organization and exercise of the ethologist profession. In this Law project, the professional competencies of an ethologist (which can be a graduate of biology or psychology studies) are summarized as the abilities to observe and analyze the behavior of all animal species not only in relation to the natural environment but also in relation to a diversity of contexts involving the interactions with human individuals and society, aiming to conserve the biodiversity and to assess, manage, and prevent problematic HAIs. According to the Law project proposal, “an ethologist is the holder of an official qualification at master’s and/or doctoral level in the field of animal behavior, respectively in the field of human-animal interactions” [27]. The law project proposal was approved for discussion at the level of specific commissions in the Romanian Senate. As one can notice, in this law project in Romania, the definition of the ethologist refers not only to the abilities to scientifically study the behaviors of animals under natural conditions from the perspective of biological mechanisms and evolutionary adaptive functions but also to the dimension of human-animal interactions, which defines the anthrozoology field. We assume that the intention behind this broader definition of the ethologist was to open the access to this profession to graduates in psychology, which is a very well represented HEI’s area of specialization in Romania, as well as to encourage new interdisciplinary academic programs to address both the animal and the human behavioral aspects (including emotions and cognitions) when dealing with problematic HAIs. While several conservation of biodiversity master’s degree programs exist in Romania, hosted by public and private HEIs usually within the specializations of ecology or biology, none of these programs include subjects addressing HAIs from a multidimensional psychological perspective, e.g., do not offer the graduates the skills to use psychometric instruments for assessing the motivational factors behind specific negative human-animal interactions.

Taking into account the intensive participation in the last two decades of the NGOs in Romania in addressing problematic HAIs and the abovementioned promising legislative changes in the area of animal welfare, the objective of this study was to use a qualitative approach (thematic content analysis of written reflections) to investigate the perceptions and the support of several animal protection and wildlife conservation NGOs in Romania regarding the opportunity of foundation, for the first time in the academic history of the country, of a specialized master’s degree in ethology and human-animal interactions. The NGOs included in this qualitative study have a history of collaboration in research and/or community-oriented events with the hosting HEI of the EHAI program, which is the University of Agricultural Sciences and Veterinary Medicine in Cluj-Napoca, Romania. We are fully aware that the number of the NGOs participating in the study is a small one and that the inclusion criteria are based on convenience, i.e., previous collaboration with the host university. However, the nature of this qualitative study is an exploratory one, aiming to generate data concerning the needs of community representatives in the fields of animal protection and wildlife conservation in Romania, as well as to identify potential ways to link these needs with the curricular content of the proposed master’s degree program and with the students educational and professional activities, in the direction of fostering their community engagement through collaboration with NGOs during the program and in the future. There is a growing recognition of the usefulness of the qualitative research (e.g., content analysis procedures) in many fields, including social and medical sciences, in providing valuable insights on the voices of individuals’ and organizations’ “life worlds” [28,29]. 

The two research questions addressed in this study are

(1) What are the needs of the animal protection and wildlife conservation NGOs in Romania that could be addressed by the first professional master’s program in ethology and human-animal interactions?

(2) What are the expectations regarding the roles of the graduates of the EHAI program on the collaboration with the NGOs in addressing common problems in the area of human-animal interactions in Romania?

## 2. Materials and Methods

### 2.1. Data Collection

Between October-November 2020, written reflections were collected by e-mail from six representatives of some of the most active NGOs in the areas of animal protection and wildlife conservation in Romania. The inclusion criteria consisted in previous collaborations with the host HEI in the field of student community engagement and/or in the field of participatory research. A number of 10 NGOs were initially contacted, but only 6 NGOs returned their written reflections in the timeframe provided, i.e., 3 weeks from the moment of receiving the message. The written reflections were collected in the form of support letters for the development and implementation of the EHAI master’s degree program in Romania). The representatives of the NGOs were asked to organize their support letters (one to two pages) in the following structure: (1) to provide a brief description of their main objectives and activities; (2) based on their own experience in the field, to provide a presentation of the problematic situations in Romania (animal protection, wildlife conservation, and HAIs) and needs that could be addressed by the EHAI master’s degree; and (3) to reflect on how they envisage the collaboration with the future graduates of the EHAI program in addressing the identified needs. Along with the abovementioned instructions, an introductory text was sent to the NGOs’ representatives with a summary of the proposed curricular content (names and short descriptions of the subjects) of the master’s degree program for 2 academic years. Participants were informed that the support letters (with the names of the NGOs) would be used for qualitative analysis and official publications. The support letters were provided in Romanian language and translated in English by the authors. 

### 2.2. Participants

A number of six directors of Romanian NGOs with local, national, and international activities participated in the qualitative study. All the participating NGOs are familiar with the academic profile of the host HEI, and they were involved over the years in joint community-oriented programs and participatory research projects. Below we present the short descriptions of the participating NGOs, such as year of foundation, location, representative activities, and the domain of activities: Animal Protection (AP), Environmental (E), or both.

- iCare is a local animal protection NGO with international connections, founded in 2014 in Cluj-Napoca, Romania. The iCare’s objectives are sheltering stray animals in a proper environment, ensuring that their basic needs are met, promoting of responsible pet ownership, conducting trap-neuter-return programs for community cats and informing the general public about the benefits of peaceful coexistence between humans and animals. Type of NGO: AP.
- Nuca is an animal protection NGO founded in 2012 in Cluj-Napoca, Romania (note: Nuca is note an acronym but a name chosen in the memory of the founder’s pet). Nuca’s activities consists in organizing sterilization and medical care campaigns for animals in community, in collaboration with veterinarians, by assuring accessible costs to low-income persons owning dogs and cats. Several awareness events are organized by Nuca NGO multiple times per year. Type of NGO: AP.
- Transylvania Animal Care (TAC Social) was founded in 2015 in Cluj-Napoca, Romania, set up both as an NGO and as a veterinary medicine business. As NGO, TAC social subsidizes spay and neuter surgery and veterinary care for animals brought in by their own volunteers, but also by other NGOs working for stray dogs and community cats, as well as for low-income pet owners. As a veterinary practice, TAC offers highly affordable veterinary services to animals (mainly dogs and cats) in Cluj-Napoca and around. Type of NGO: AP.
- Luana’s Dream Foundation (LDF) was founded in 2014 in Popesti-Leordeni, Romania. LDF is a non-profit organization that conducts medical care programs for injured wild animals, sterilization campaigns for controlling the reproduction of domestic animals with and without owners, as well as environment-oriented activities, such as planting and greening campaigns. LDF is hosting the Wildlife Rescue and Rehabilitation Centre, which, besides the veterinary medical care unit, is constantly offering volunteer openings and awareness education programs to community, especially to children and adolescents. Type of NGO: AP&E.
- Animal Society Romania (former Vierpfoten Romania) is active since 2003 in Bucharest, Romania. The activities comprise humane and sustainable management of dog and cat populations through sterilization campaigns, as well as information and awareness actions on the importance of sterilization, vaccination, and responsible adoption. In addition, the NGO includes a Centre for Animal Assisted Therapy and Activities with former community dogs, being the only center of this type in Romania. Volunteering openings and animal-assisted educational programs for children are regularly organized by the Animal Society Romania NGO. Type of NGO: AP.
- WWF Romania is active since 2006 in Romania, where it conducts several programs to protect the wild environment in the Carpathian Mountains and along the Danube: protected areas, forests, brown bears, bisons, Danube Delta, sturgeons etc. In addition, WWF Romania is involved in the stimulation of the transition to the green economy and in offering environmental education programs for young people in Romania. Type of NGO: E.

### 2.3. Data Analysis 

The qualitative data analysis of the raw data (i.e., the written reflections provided by the six directors of the NGOs) was performed following the hands-on guide to doing thematic content analysis [28]. According to Erlingson and Brysiewicz [28], the objective of the qualitative content analysis is to systematically transform a large amount of text (written reflections, verbatim transcribed oral interviews, etc.), which is considered the raw data, into a concise and organized summary of the key results. The following steps were performed: 1. Reading the written reflections to gain a general understanding of what the participants are expressing, 2. Condensation of the text by division into meaning units (codes and sub-codes), 3. Grouping the codes into categories (i.e., a category is formed by grouping together those codes that are related to each other through their content or context) and themes (i.e., a theme can be seen as expressing an underlying meaning found in two or more categories). The written reflections were manually condensed by Alina Simona Rusu (ASR), followed by discussion and agreement among the three authors regarding the codes, as well as the categories and the identified themes. The two identified themes (Table 1 and Table 2) were in line with the two research questions. 

## 3. Results

### 3.1. Theme 1—Concerns Identified by the NGOs over Animal Welfare and HAIs in Romania

#### 3.1.1. Concerns over Stray Animals

All the representatives of the NGOs participating in the study, including the two ones with focus on wildlife conservation and environment protection, indicated the problematic situation of community animal (dogs and cats) number and management in the urban and rural areas of Romania, as well as the high rate of abandonment and of adoption of animals from private and public shelters. The concerns over stray animals were presented by the respondents in connection to the other main codes, especially with the lack of education regarding human-animal interactions:

*“The high number of animals in the streets is influenced by the size of abandonment and the size of absorption of shelter animals in the population, both dimensions being determined by the level of education and understanding of human-animal relations.”*

(AP01, November 2020)

#### 3.1.2. Lack of Education of General Population on HAI and Animal Welfare

The main code “lack of education” appeared associated with various attributes, such as “serious,” “problematic,” “with severe consequences,” etc. The lack of education regarding responsible ownership, treatment of companion, and wild animals, appeared in all the six written reflections with a frequency of 2–6 times, being associated with several categories of factors, such as age (kindergarten children, adolescents, and adults), statuses and roles (parents, animal owners, school staff, tourists, and zoo visitors), and locations (urban, rural, and protected areas).

*“Over the ten years of activity in the field, we have faced an overwhelming proportion of a very uneducated public on basic notions of animal behavior, a phenomenon transposed by a precarious understanding of the legal and moral aspects of abandonment and aggression toward animals…”*

(AP02, November 2020)

*“We are trying to achieve our goals by providing as much as we can educational campaigns in kindergartens and schools. Informing and educating the general population about human-animal interactions is essential in creating a society in which respect for all living beings and protection of the environment are core values.”*

(AP01, October 2020)

*“The Romanian society has, as a whole, a precarious relationship with animals, often marked by a serious lack of information, knowledge and contemporary skills, and, in many cases, by superstitions, beliefs and local traditions; all of these, plus the ignorance, affect not only animal welfare as an immediate reality, but also the human and community quality of life.”*

(AP03, October 2020)

#### 3.1.3. Maltreatment of Community and Companion Animals

The codes identified within the maltreatment of community and companion animals’ category were reflected in the written reflections of five of the NGOs’ representatives. As stated above, the forms of cruelty towards animals, such as aggression, extreme violence, and the neglect, were always presented in association with the lack of adequate education in the area of HAIs and animal welfare legislation, but also with the ignorance of the existing local, national, and European legislative measures. The maltreatment of the community animals was placed by one respondent under the umbrella of human-animal conflict:

*“…This type of conflict situations, that usually ends with tragic consequences for animals (from abandonment to cases of extreme violence), occurs due to ignorance, disobedience and non-application of the laws.”*

(AP&E01, November 2020)

#### 3.1.4. Negative Human-Animal Interactions

The codes within this category referred to both domesticated animals (companion and community animals, especially dogs and cats) and wild animals. Particularly, the two NGOs with activities in the areas of wildlife conservation and rehabilitation indicated several conflicting situations between wild animals and humans, pointing out the potential causes, such as intensification of human activities, urbanization, and lack of information and educational program for the general public in regards to the impact of human activities on the wild animals, their habitats and the environment.

*“From the point of view of wildlife in Romania, over the last 5 years, we have noticed that, with the excessive urbanization, some wild species have managed to find an ecological niche in the anthropic environment. However, these animals often suffer from a lack of information of the citizens about the needs of these species and the appropriate ways to interact with them.”*

(AP&E01, November 2020)

*“…The number of wild-human animal problematic situations in Romania is constantly increasing, which amplifies the pressure on animals and often leads to radical measures against them.”*

(E01, October 2020)

#### 3.1.5. Lack of Professional Specializations in HAI

The lack of professional specializations in human-animal interactions was mentioned in several forms by all of the participants, some of them even pointing the urge of providing modern programs to train specialists in the field in order to prevent and manage the negative HAIs. Affective reactions, such as sadness and disappointment associated with the pauperism of professionals in ethology and HAI, were reflected in most of the statements:

*“Unfortunately, the background of our national bad tradition of interacting with animals is based on two factors: the social and cultural habits of interactions between humans and animals and on the missing of authentic specialists to mentor these types of interactions. If the first factor can not be easily repaired, it is urgent to train specialists who can change the present and shape the future in this field like in other modern countries.”*

(AP02, October 2020)

*“…The lack of specialists and professional branches in Romania to understand in depth both the behavior and the vital needs of animals, especially in relation to the human presence, as well as aspects of legislative framework that should more efficiently protect animals, unfortunately creates many instances of conflict or ignorance, hence, animals often have suffered.”*

(AP01, November 2020)

#### 3.1.6. Concerns over Common Global Issues

The content analysis of the written reflections provided by the six NGOs depicted a series of common concerns over common global issues (Table 1), in some cases associated with the need of specialized programs in the field of animal welfare and HAI. Three NGOs representatives referred to the degradation of the planet’s natural environment, to the decrease in the number of wild populations of animals, climate change, and irrational food consumption by humans, while the increasing zoonotic risks that can lead to pandemics was mentioned by two of the NGOs, i.e., the ones with areas of activities in the field of wildlife conservation and rehabilitation.

*“According to the Living Planet Index, from 1970 to 2016, there was an average decrease of 68% in size at global level of the wild population of mammals, birds, amphibians and fish, which indicates that, on one hand, we do not know how to appreciate the value and importance of these species and on the other hand we do not have enough trained specialists to prevent these situations.”*

(E01, October 2020)

*“In the context of the more frequent and severe zoonosis than can lead to pandemics, but also of climate change, human-animal interactions is expected to become an increasingly delicate but crucially important aspect.”*

(AP03, October 2020)

#### 3.1.7. Concerns over National Nature Conservation

The concerns over wildlife and nature conservation at national level were expressed by three of the NGOs, in two cases in association with national assets, such as the existence of numerous active NGOs that are in a constant state of readiness to intervene in solving problematic and crises situations (although it is indicated that the objectives are to prevent these situations through law reinforcement strategies and through the involvement of well-prepared specialists in the HAI field) and the high natural diversity of Romania (Table 1).

*“Often chaotic economic development, with projects that do not always and/or completely take into account the negative impact they may have on wildlife and environment, it is still a major and complex threat in Romania, given that our country is (still) among the richest in natural diversity of the European Union members.”*

(E01, October 2020)

*“In the present year, there are dedicated public institutions and numerous NGOs in Romania with the best intentions in regulating the relations between humans, animals and the environment.”*

(AP04, November 2020)

*“National capacity building in the field of wildlife and environment conservation is a constant concern for us, without which we can not talk about viable long-term results and sustainability.”*

(AP&E01, October 2020)

### 3.2. Theme 2—Expectations Regarding the Roles of the Graduates of the EHAI Master’s Program

#### 3.2.1. Agents for Change towards a Responsible Community

As indicated in Table 2, the graduates of the EHAI master’s degree program are expected to contribute through their personal and professional skills and activities to the development of responsible community in terms of human-animal and human-environment interactions, by becoming active participants in creating a society in which the respect for all living beings and the protection of the environment are core values. In line with this, literature in the field of Humane Education in universities indicates that, besides the curricular content and the activities in which the students are involved in relation to the community needs over the academic years, an important role in developing agents for change is represented by the institutional sustainable culture itself [5], which can facilitate the internalization of these core values by the staff and by the students. All the participating NGOs pointed out the need for openness and readiness of the graduates to educate and increase the awareness of general public about significant aspects of responsible human-animal interactions in the direction of conflict preventions.

*“In the absence of a clear legislation framework on owning an animal and managing community animals, it is education that can provide agents for change for a responsible community and that can contribute to solving the inherent problems of community animals.”*

(AP04, October 2020)

*“We want the general public to have access to well trained specialists that can be a valuable source of local information in regards to the human-animal interactions and conflicts.”*

(AP01, November 2020)

#### 3.2.2. Problem Solvers and Public Policy-Makers

The two codes “problem solvers” and “public policy-makers” were mentioned either individually (5 times) or in the same statement (one time), always in association with concepts such as coexistence of humans with animals, education and attention to the needs of animals and the wellbeing of humans and of the society.

*“We believe that the graduates trained through this program will be able to contribute in the future in an essential way to solving current problems and that they will find sustainable solutions for a better coexistence with animals, which will increase the welfare of animals and the wellbeing of humans.”*

(AP02, November 2020)

*“The state of problems that our teams deals regularly in its veterinary medical work, advocacy and education of the people in the community, can only be changed by academically trained and certified specialists in ethology and human-animal interactions, who can become the competent educators of our civil and administrative communities, public policy makers and mentors of the school population at any level, which is currently deprived of structured and modern education in human-animal interactions.”*

(AP&E01, October 2020)

#### 3.2.3. Providers of Competence-Based Expertise

The codes included in this category provided valuable information regarding the competencies and the learning outcomes that are expected by the representatives of the communities (NGOs) from the EHAI master’s degree program. Although the components of the One Health approach are mentioned by all the NGOs in their statements regarding the professional expectancies of the EHAI master’s graduates, the concept itself appears in one out of the six written reflections collected in this study.

*“We, as organization, are working for almost 20 years in the field of animal protection, and we support the development of programs to academically train professionals who can make an impact within the large concept of One Health, that underlines and strengthens the human-animal responsible interactions.”*

(AP04, November 2020)

## 4. Discussion

This study aimed to perform an exploratory qualitative analysis of the reflections of six animal protection and wildlife conservation NGOs in Romania regarding the development of the first specialized master’s degree program in ethology and human-animal interactions (EHAI) in the country. The reflections were expressed in relation to the identified needs based on each NGO’s experience in the field, as well as the expectations in terms of collaboration with the graduates of such a program in addressing the common problems in the areas of animal welfare and human-animal interactions.

Following the qualitative content analysis of the written reflections, two themes were identified, which indicate that, although the participants did not provide explicit answers to the research questions, their reflections allow for interpretations and depiction of implicit meanings that can be further addressed either in the co-construction of the curricular content and/or in planning educational and service-based activities for students in partnership with the NGOs. 

The first theme is represented by the *concerns identified by the NGOs over the animal welfare and HAIs in Romania*, and it includes seven categories: concerns over stray animals, lack of education of general population on HAIs and animal welfare, maltreatment of community and companion animals, negative human-animal interactions, lack of professional specializations in HAIs, concerns over common global issues, and concerns over national nature conservation. One way of dealing with the feedback regarding these categories of concerns is to address them through the curricular content of the disciplines included in the EHAI master’s degree program. It is important to mention that the curricular content of the EHAI program was planned after reviewing the structure of similar master’s degree programs in the European space and around the world, as well as based on consultation with international collaborators in the areas of animal behavior, anthrozoology, and wildlife conservation. The graduates of this program, through the disciplines included in the curriculum, are expected to gain in-depth knowledge of grounded theories and models that explain and support not only the investigation of the natural behaviors of animals, biological stress in various conditions of captivity, and HAIs but also skills on collection and analysis of behavioral and psych-physiological data. Another way of addressing the identified concerns in relation to the curricular content is to invite representatives of NGOs to present case studies and co-reflect with the students on the causes of the problematic situations and potential solutions of management and prevention. 

A particular category is represented by the concern over the lack of education of general population on HAIs and animal welfare, which was pointed out by all the participants, and it was usually presented in a casual relation with a diverse array of negative human-animal interactions. This concern was interpreted in connection with the findings of the second research questions regarding the expected roles of the EHAI master’s degree graduates to be providers of education towards responsible and sustainable HAIs. We consider that this concern points towards the need to offer the students of the EHAI program the knowledge and the skills to educate others, especially children, as indicated by all the NGOs.

The EHAI master’s degree program is proposed for use in tertiary education settings, meaning that it will include mature students that most likely have undergone a pre-service teacher training, which in Romania is usually offered at undergraduate level. The students that did not receive pre-service teaching training at undergraduate level can decide later on to enroll in didactic training modules during the master’s degree program. However, this decision is an individual one, so it does not guarantee that all the students will have the formal competencies to be involved in educational programs. This particular concern motivates us to foster the inclusion of educational theories and methods in the curricula of the disciplines addressing the animal welfare and HAIs in a variety of contexts. Cases of successful educational programs, particularly of those that involve students, will be presented as examples of good practice. Additionally, joint projects between the students of the EHAI master’s degree program and students of Educational Sciences will be encouraged, in which students can design together and exchange experiences regarding the ways of community education towards responsible and sustainable HAIs.

The second identified theme refers to *the expectations regarding the roles of the graduates of the EHAI master’s program* and it includes the following four categories: agents for change towards a responsible community, problem solvers, public policy-makers, and providers of competence-based expertise. As indicated in the second research question of this exploratory qualitative study, we aimed to identify the expectations of the participants regarding the roles of the graduates of the EHAI program on the collaboration with NGOs in addressing common problems in the area of human-animal interactions in Romania. Even though the findings reflect only the expected roles of the graduates, we decided to interpret them through the frame of the research question, i.e., how can the master program offer the ground to develop the expected roles of the EHAI students through collaboration with NGOs during the program and following their graduation.

The content analysis of the NGOs’ reflections indicated that the expected role that appeared first in the text of the support letters was the one referring to the graduates as agents for change towards a responsible community, followed by the role of problem solvers and public policy-makers. As recently indicated by international experts in Humane Education in Higher Education, an important part of capacity building is “training for change agents of those who will be responsible for facilitating change” not only in the organization but also in the community and in the society [14,30]. The fact that all the participating NGOs have indicated the need to academically train agents for change towards a responsible society have motivated us to focus on the importance of Service-Learning as a method to respond to this need, by including the valuable role that the collaboration with the animal protection and wildlife conservation NGOs can bring into this process. Service-Learning (SL) is generally defined as an innovative educational strategy that combines experiential learning with services addressing community needs, aiming to develop civic-oriented attitudes and behaviors of the students [13,31]. While SL is still a novelty in terms of educational practice in HEIs in Romania [32,33,34], promising steps are being made in the direction of building awareness towards the benefits of SL at levels of students, teaching staff, institution climate and representatives of the community. These steps refer to collaborative projects within the Erasmus + frame, which often lead to development of valuable networks based on exchange of good practices, as well as to training programs and manuals for students and academics [35]. SL is considered one of the most valuable and meaningful tools that can be used by HEIs to achieve their “third mission” [36], which is defined as “… *the generation, use, application and exploitation of knowledge and other university capabilities outside academic environments*” [37,38]. In line with the expressed roles of the EHAI graduates, we aim to include SL educational strategy both as a component of several disciplines (e.g., credited individual or group projects) and as an elective discipline, which can be chosen by the students that are motivated to deepen their knowledge and abilities in designing and implementing diverse SL projects. Official forms of collaboration with the NGOs willing to foster the civic engagement of students in addressing HAIs-related community need will be managed at institutional level. Moreover, in response to the expected roles of problem solvers and public policy-makers, we aim to implement the method of rotation SL technique [39], which allows students to be engaged in several NGOs during one SL project, thus experiencing a diversity of challenges and contexts of human-animal interactions. 

Another aspect supporting our motivation to include SL in the structure of the EHAI master’s degree program, which is in line with the expectations of the roles of the graduates expressed by the six NGOs, is the positive impact that SL programs can have on the personal development of students, their prosocial attitudes, civic action propensity, political awareness, appreciation of diverse attitudes, and the feeling of being able to “make a difference” in the community [13,40,41]. In addition, many students are reported to be more likely to volunteer after SL experiences during their college education, and some of them are later on employed by the NGOs that provided the SL opportunities; [13,34,42,43]). 

We do acknowledge that a limitation of this qualitative exploratory study is the potential positive bias in the reflections collected from the representatives of the six participating NGOs, with whom the host institution had previously collaborated in community-oriented events and/or participatory research projects, as well as the fact that the four non-responses may reflect not only the lack of time of the representatives of those particular NGOs but also a neutral and/or non-supportive position toward the initiative of developing the EHAI master’s degree program.

## 5. Conclusions

In conclusion, the content analysis of the letters of support provided by the six NGOs for the development of the first master program in Romania in ethology and human-animal interactions has offered valuable insights in the direction of finding meaningful ways for the co-construction of the curricular content and of the educational and professional activities of the students, which are expected to become educators of the community, problem solvers, and agents for change towards a responsible community in relation to animals, people, and environment. The solutions proposed by us to address the expressed concerns over the animal welfare, wildlife conservations and HAIs in Romania, as well as the expected roles of the EHAI graduates, were elaborated within the community engagement approach, thus interpreting the findings from the perspective of the formulated research questions. According to the last report of the Network of Experts Working on the Social Dimension of Education and Training [44], community engagement refers to “the range of ways in which university staff, students and management interact with external communities in mutually beneficial ways, either as part of teaching and research or as part of other projects and joint initiatives.” We are fully aware that the small sample of the participating NGOs does not allow for the generalization of the results to other existing NGOs in Romania. However, the themes and the categories identified in the current qualitative analysis might serve as a starting point for developing a more elaborated survey to assess the needs of animal protection and wildlife conservation community representatives in Romania in the context of collaboration with the academic institutions.

## Figures and Tables

**Table 1:** Presentation of the categories and the codes corresponding to the theme “Concerns identified by the NGOs over animal welfare and HAIs (human-animal interactions) in Romania,” which were identified in the course of content analysis of the written reflections of the representatives of the six NGOs participating in the study.

Theme	Categories	Codes
Concerns identified by the NGOs over the animal welfare and HAI in Romania	Concerns over stray animals	High number of stray animals Low rate of adoption of animals from public and private shelters High rate of abandonment in urban and rural areas
	Lack of education of general population on HAIs and animal welfare	Overwhelming proportion of uneducated public on basic notions of animal behavior Poor understanding of the legal and moral aspects of abandonment Poor preparation for long-term responsible adoption Actions towards animals based on superstitions and cultural habits Ignorance of the needs of animals Ignorance of the animal welfare legislation Lack of modern HAI educational approach for children Serious lack of HAI knowledge and contemporary skills
	Maltreatment of community and companion animals	Aggression and killing of community animals Situations of cruelty and ill-treatment of animals, from abandonment to cases of extreme violence Precarious relationship with companion animals
	Negative human-animal interactions	Negative HAI situations with tragic consequences for animals Lack of information among the general public regarding the urbanization effects on wild animals Pressure of the intensification of human activities on wild animals and their habitats Increasing number of wild negative HAIs in urban and peri-urban areas Radical measures against wild animals that are considered problematic Excessive urbanization and destruction of wildlife habitat Increasing number of injured wild animals
	Lack of professional specializations in HAI	Lack of specialists to understand in depth the behavior and the needs of animals, especially in relation to human presence Lack of specialization programs for the study of animal life in relation to human life in the current global context
	Concerns over common global issues	Degradation of the planet’s natural environment Decrease the size of wild populations of mammals, birds, amphibians, reptiles, and fish Irrational food consumption by humans Frequent and severe zoonoses that can lead to pandemics Climate change
	Concerns over national nature conservation	Lack of a coherent strategy for nature conservation Economic development projects not always taking into account the negative impact on the environment

**Table 2:** Presentation of the categories and the codes corresponding to the theme “Expectations regarding the roles of the Ethology & Human-Animal Interaction (EHAI) master’s degree graduates on the collaboration with the NGOs in addressing common problems in the area of Human-Animal Interactions (HAIs) in Romania.”.

Theme	Categories	Codes
Expectations regarding the roles of the graduates of the EHAI master’s program	Agents for change towards a responsible community	Contribute to the development of a responsible community General public will have access to specialists Be a valuable resource of local information Be involved in creating a society in which respect for all living things and protection of the environment are core values. Make possible the human-animal coexistence and interaction in the most harmonious possible ways Become trained specialists who can change the present of the country
	Problem solvers	Contribute in the future in a meaningful way to solving and preventing current problems related to all types of negative HAIs Find sustainable solutions for a better coexistence with animals, which will increase their welfare. Come up with concrete/specific solutions regarding human-animal conflicts Respond to the needs of both society and animals
	Public policy-makers	Contribute to the national and international public policies regarding animal welfare and HAIs Help the country to make progress towards a sustainable future through realistic public policies
	Providers of competence-based expertise	Offer their expertise where it is needed, based on a formal specialization in the field of ethology, which is under represented in the Romanian academic community Perform competence-based work, including education and advocacy Represent and support in a professional manner the field of EHAI program Provide expertise within the concept of “One Health”

## Data Availability

The data presented in this study are available on request from the corresponding author.
